# Well-Water Consumption and Parkinson’s Disease in Rural California

**DOI:** 10.1289/ehp.0900852

**Published:** 2009-07-31

**Authors:** Nicole M. Gatto, Myles Cockburn, Jeff Bronstein, Angelika D. Manthripragada, Beate Ritz

**Affiliations:** 1 Department of Epidemiology, University of California–Los Angeles, Los Angeles, California, USA; 2 Department of Preventive Medicine, University of Southern California, Los Angeles, California, USA; 3 Department of Neurology and; 4 Department of Environmental Health Sciences, University of California–Los Angeles, Los Angeles, California, USA

**Keywords:** agriculture, contamination, Parkinson’s, pesticide, well water

## Abstract

**Introduction:**

Investigators have hypothesized that consuming pesticide-contaminated well water plays a role in Parkinson’s disease (PD), and several previous epidemiologic studies support this hypothesis.

**Objectives:**

We investigated whether consuming water from private wells located in areas with documented historical pesticide use was associated with an increased risk of PD.

**Methods:**

We employed a geographic information system (GIS)–based model to estimate potential well-water contamination from agricultural pesticides among 368 cases and 341 population controls enrolled in the Parkinson’s Environment and Genes Study (PEG). We separately examined 6 pesticides (diazinon, chlorpyrifos, propargite, paraquat, dimethoate, and methomyl) from among 26 chemicals selected for their potential to pollute groundwater or for their interest in PD, and because at least 10% of our population was exposed to them.

**Results:**

Cases were more likely to have consumed private well water and to have consumed it on average 4.3 years longer than controls (*p* = 0.02). High levels of possible well-water contamination with methomyl [odds ratio (OR) = 1.67; 95% confidence interval (CI), 1.00–2.78]), chlorpyrifos (OR = 1.87; 95% CI, 1.05–3.31), and propargite (OR = 1.92; 95% CI, 1.15–3.20) resulted in approximately 70–90% increases in relative risk of PD. Adjusting for ambient pesticide exposures only slightly attenuated these increases. Exposure to a higher number of water-soluble pesticides and organophosphate pesticides also increased the relative risk of PD.

**Conclusion:**

Our study, the first to use agricultural pesticide application records, adds evidence that consuming well water presumably contaminated with pesticides may play a role in the etiology of PD.

Multiple lines of evidence link pesticides as possible contributors to the pathogenesis of Parkinson’s disease (PD). Many epidemiologic studies previously reported associations between pesticide exposure, rural living, and farming and the development of PD (Ben-Shlomo et al. 1993; Burguera et al. 1992; Morano et al. 1994; Svenson et al. 1993). A number of animal studies have also supported a potential etiologic role of pesticides in PD (Betarbet et al. 2000; Norris et al. 2007; Sherer et al. 2001; Thiruchelvam et al. 2000, 2002, 2003). The ingestion of contaminated drinking water is a potentially important vehicle for pesticide exposure in human populations. Epidemiologic studies previously examined links between well-water consumption and PD, and most provided support for positive associations (Firestone et al. 2005; Hancock et al. 2007; Nuti et al. 2004; Priyadarshi et al. 2001; Wright and Keller-Byrne 2005). All existing studies have relied on self-reports of well-water consumption and used broad ever/never exposure categories. Such studies may have suffered from recall bias and exposure misclassification. Most importantly, no study to date has attempted to specify pesticide exposure levels by assessing or estimating the contamination of well water with specific pesticides.

Although the Safe Drinking Water Act of 1974 was passed to regulate the public drinking water supply, private wells in the United States are not subject to the same regulations and thus are not similarly monitored or held to the same water-quality standards as are public systems. Furthermore, many private wells are dug or driven at shallow depths (i.e., < 15–20 yards), which place them at risk of being contaminated by land activities such as pesticide applications in the vicinity of a well (U.S. Environmental Protection Agency 2002). Pesticides may move from their initial intended area of application. Investigators have shown that measurable concentrations of pesticides have been detected in air, water, plants, and animals up to several hundred meters from the application sites (Chester and Ward 1984; Currier et al. 1982; MacCollom et al. 1986), emphasizing the need for methods to assess environmental exposures due to drift and contamination of soil, air, and water in agricultural communities. Geographic information system (GIS)–based methods of assessing exposures to pesticides may prove an effective solution when comprehensive pesticide-application data exist. We developed and employed a GIS**–**based exposure assessment tool to estimate pesticide exposures from applications to agricultural crops using data from California pesticide use reports (PURs), land-use maps, and geocoded residential historical addresses (Goldberg et al. 2007). We combined this information with data on well-water consumption collected in interviews with study participants to estimate exposure to potentially pesticide–contaminated well water. In this study, we investigated whether consumption of water from private wells located in areas with documented historical agricultural pesticide use was associated with an increased risk of PD among residents of the Central Valley of California, well known for its intensive agricultural activities.

## Materials and Methods

### Study population

We used a population-based approach for recruiting cases and controls from a largely agricultural population in California. Details are provided elsewhere (Kang et al. 2005). Briefly, study subjects were recruited between January 2001 and January 2007, resided in Fresno, Tulare, or Kern County, and had lived in California for at least 5 years before diagnosis or interview. Cases were recruited within 3 years of diagnosis, were not in the last stages of a terminal illness, agreed to participate, and were confirmed as having clinically probable or possible PD by a University of California–Los Angeles (UCLA) movement disorder specialist. A diagnosis of clinically probable or possible PD was confirmed if patients met the following criteria: 1) manifestation of at least two of the following symptoms: resting tremor, bradykinesia, or cogwheel rigidity; 2) no suggestion of a parkinsonian syndrome due to trauma, brain tumor, infection, cerebrovascular disease, other known neurologic disease, or treatment with dopamine-blocking or dopamine-depleting agents; 3) no atypical features such as prominent oculomotor palsy, cerebellar signs, vocal cord paresis, severe orthostatic hypotension, pyramidal signs, amyotrophy, or limb apraxia; 4) asymmetric onset; and 5) if treatment with levodopa had been initiated, symptomatic improvement after treatment. Probable cases met criteria 1 through 4 with or without treatment (criterion 5). Possible cases had at least one sign from criterion 1 and fulfilled criteria 2 and 3. Although sometimes included in criterion 1, postural reflex impairment was excluded because it usually occurs late in PD and may typically occur early in other parkinsonian disorders, such as multiple system atrophy and vascular parkinsonism. Of the 31 practicing local neurologists who provided care for PD patients, 28 (90%) assisted in recruiting cases for this study. We solicited collaboration from Kaiser Permanente in Oakland, California, USA, to obtain data from the medical offices in Fresno, California, USA, Kern Medical Center in Bakersfield, California, USA, Visalia Medical Clinic in Visalia, California, USA, the Veteran’s Administration in Fresno, California, USA, PD support groups in all three counties, as well as local newspapers and radio stations that broadcast public service announcements. Of the 1,167 PD cases who were initially invited to participate in the study, 604 were not eligible: 397 whose diagnosis date fell outside the 3-year range before contact, 51 who denied having received a PD diagnosis, 134 who lived outside the tricounty area, and 22 who were too ill to participate. Of the 563 eligible cases, 473 (84%) were examined by a UCLA movement disorder specialist at least once and confirmed as having clinically “probable” or “possible” PD; the remaining 90 potential cases could not be examined or interviewed (54% withdrew, 32% were too ill or had died, and 14% moved out of the area before the exam or did not honor a scheduled appointment). We examined but excluded another 93 patients because of other causes of Parkinsonism and 1 case whose diagnosis was still not confirmed at the time of this analysis. A total of 379 cases were included in the study; of these, 368 provided all information needed for the analyses.

Controls who were > 65 years of age were identified from Medicare lists in 2001, but because of the implementation of the Health Insurance Portability and Accountability Act (1996), which provides federal protections for personal health information held by covered entities, we were prohibited from using Medicare enrollees as controls. More than 70% of our controls were recruited from randomly selected tax assessor residential units (parcels) in each of the three counties. We mailed letters of invitation to a random selection of residential living units and also attempted to identify head-of-household names and telephone numbers for these parcels using the services of marketing companies and Internet searches. We contacted 1,212 potential controls by mail and by phone for eligibility screening. Controls were eligible to participate if they *a*) did not have PD, *b*) were at least 35 years of age, *c*) were currently residing primarily in one of the three counties, and *d*) had lived in California for at least 5 years before the screening. Only one person per household was allowed to enroll. A total of 457 controls were ineligible: 409 were too young, 44 were terminally ill, and 4 primarily resided outside of the study area. Of the 755 eligible population controls, 409 (54%) declined participation, were too ill to honor an appointment, or moved out of the area before their interview. A total of 346 (46%) controls were enrolled, and 341 provided all information needed for the analyses.

### Data collection

Trained interviewers who were blind to case/control status conducted structured telephone interviews to obtain demographic and exposure data from study participants. Detailed questionnaires that queried subjects for their lifetime residential addresses were mailed to subjects in advance of their interview and were reviewed in person or over the phone; we asked about the type of water supply at each address (public supply, private well, filtered water, bottled water, other). All subjects provided informed consent, and the UCLA Human Subjects Committee approved all study protocols.

### Pesticide exposure assessment

We geocoded lifetime residential addresses and estimated ambient pesticide application rates from agricultural uses (in pounds per acre per year) within 500 m of each subject’s home by using a validated GIS-based system that combined California PUR data and land-use maps (Goldberg et al. 2007; Rull and Ritz 2003). We estimated ambient exposure for all historical residential addresses inhabited between 1974 and 1999, the period covered by the PUR data. A technical discussion of our GIS-based approach is provided elsewhere (Goldberg et al. 2007). Here we briefly summarize the data sources and exposure modeling process.

### Geocoding residential addresses

Addresses were automatically geocoded (NavTeq 2006) to TigerLine files (U.S. Census Bureau 2009) and then manually resolved in a multistep process similar to that described by McElroy et al. (2003). The resulting locations were recorded along with the relevant year range of residence and matched to the appropriate year-specific PUR and land-use data (see below). For our GIS model we relied on addresses in Fresno, Kern, and Tulare counties (tricounty area) between 1974 and 1999 inclusive. Of the 9,568 total residential years contributed by cases (26 years × 368 cases), 7,266 (76%) years were spent at addresses within the tricounty area, compared with 6,514 (73%) of 8,866 years contributed by controls (26 years × 341 controls). We geocoded these residential addresses during the period with similar precision for cases and controls; both groups spent 88% of their respective residential years at addresses we considered to be mapped with high precision, such as at the level of a residential parcel, street address, or street intersection rather than a ZIP code or city centroid.

### Pesticides use reporting and land-use maps

PURs are collected by the California Department of Pesticide Regulation (CADPR) for any commercial application of restricted-use pesticides (defined as “agents with harmful environmental or toxicologic effects”) and, since 1990, all commercial uses of pesticides regardless of toxicologic profile. The location of each PUR record is referenced to the Public Land Survey System (PLSS), a nationwide grid that parcels land into sections at varying resolutions (~ 1 m^2^). Each PUR record includes the name of the pesticide’s active ingredient, the poundage applied, the crop and acreage of the field, the application method, and the date of application. Because the PUR records only link agricultural pesticide application to an entire PLSS grid section, we added information from land-use maps to more precisely locate the pesticide application, as described in detail elsewhere (Rull and Ritz 2003). The California Department of Water Resources periodically performs countywide large-scale surveys of land use and crop cover every 7–10 years, which allowed us to identify the location of specific crops within each PLSS grid section. Digital maps from more recent (1996–1999) surveys are available, and paper maps were manually digitized for earlier periods (1977–1995). The 1977 land-use survey was conducted closest in time to 1974 when PUR became available. We constructed historical electronic maps of land use and crop type, and using the PLSS grid section and crop type reported on the PUR, we allocated pesticide applications to an agricultural site to which we assigned a GIS-based location.

### Ambient pesticide exposure estimates

Pesticide application rates for individual chemicals (in pounds of active ingredient per acre) were summed across PLSS sections by year; these annual rates were then divided by the actual area within a 500-m radius around the home (i.e., “residential buffer”) (Chester and Ward 1984; MacCollom et al. 1986; McElroy et al. 2003) to represent the portion of a chemical application rate that a person might have been exposed to for the relevant years of residence. Total annual application rates were then weighted by the proportion of treated acreage in the residential buffer using land-use information, again to more accurately predict exposure. This resulted in ambient pesticide exposure estimates for each subject for each year. We also summed pesticide application rates across the 1974–1999 period to calculate a 26-year cumulative total ambient pesticide exposure estimate for each subject.

### Selection of pesticides relevant for well- water contamination

We selected pesticides from the CADPR Groundwater Protection List (CADPR 2009) that includes chemicals previously detected in California groundwater (*n* = 7), or designated as having the potential to pollute groundwater and expected to be detected in groundwater in California (*n* = 62) (CADPR 2009) based on their solubility, adsorption, or half-life. Two pesticides in the first category (simazine, diuron) and 17 pesticides from the latter category had been applied in our study areas during the period of interest. We selected four additional pesticides (chlorpyrifos, trichlorfon, propargite, dicofol) that were not named on the CADPR Groundwater Protection List but were identified by other states as being a concern to groundwater because of their high runoff or leaching potential, which was also based on their solubility, adsorption, or half-life (Cook et al. 2008). We also selected three chemicals of special interest for PD (paraquat, maneb, permethrin) but whose chemistry did not necessarily qualify them as potential groundwater contaminants (Ascherio et al. 2006; Brown et al. 2006; Miller et al. 1998). In total, 26 pesticides were selected for the analyses (Table 1).

### Pesticide exposures from private well water

Our estimates of well-water pesticide exposure were based on a combination of pesticide use and application data and self-reports of private wells as drinking water sources at a residential address. We assumed that private wells were likely to be located within 500-m residential buffers, and thus agricultural pesticides applied within this area were considered a source of potential contamination for water drawn from the private well. We used our GIS-modeled pesticide application data to determine which agent could have potentially contaminated a subject’s well water. We considered a potentially exposed residential year as each year at a residential address that fell into the 1974–1999 period and for which a private well was reported as the source of drinking water and calculated the annual level of exposure for each chemical with our GIS–PUR model. Then, we calculated a cumulative well-water exposure measure for each chemical for the entire 1974–1999 period by summing over the years a participant was presumed to have been exposed, that is the years the person had been drinking potentially contaminated well water. Thus, for the 1974–1999 period, those who lived at residences that were supplied with water from a private well were classified as possibly exposed to pesticides in well water at a level equivalent to the cumulative application rate predicted from our model for all years exposed (i.e., cumulative ambient pesticide application rate > 0). Participants were considered unexposed if they *a*) did not report private well water as their source of water for a given address during the 1974–1999 period (including 5.25% of addresses for which data on water supply was missing), *b*) reported private well water as their source of water for a given address before 1974 or after 1999, or *c*) reported private well water use during the 1974–1999 period but pesticides had not been applied in the buffer of the reported address according to our GIS model (i.e., cumulative ambient pesticide application rate = 0).

We also considered a second exposure classification that combined information about pesticide exposures from ambient sources and ingestion of well water, and compared subjects who were unexposed (no well-water use and no ambient exposure) with subjects who were ambiently exposed only [no well-water use during 1974–1999, but pesticides were applied near the residence(s)]; and ambiently exposed and well water was their source of drinking water for all or a portion of the 1974–1999 time period.

We individually examined from the list of 26 selected chemicals the 6 pesticides for which at least 10% of our population were ambiently exposed (diazinon, chlorpyrifos, propargite, paraquat, dimethoate, and methomyl). For subjects suspected of having been exposed to pesticides via well water, we created categories of any exposure versus no exposure, and high and low exposures versus no exposure (with exposure categories based on the median values of possible pesticide exposure in the well water of controls). We also examined combined exposure measures according to the number of pesticides applied from within two chemical classes in our list of 26: organophosphate (OP) pesticides (10 total; grouped as 5–10, 1–4 vs. 0) and *N*-methylcarbamate pesticides (4 total; grouped as 3–4, 1–2 vs. 0; Table 1). Similarly, we examined possible exposure to water-soluble pesticides as a group (19 total; grouped as 10–17, 1–9 vs. 0; no one was exposed to all 19) and all pesticides as a group (26 total; grouped as 12–24, 1–11 vs. 0; no one was exposed to all 26). For the combined measures (pesticide chemical classes, water-soluble pesticides, all pesticides), we followed our definition of well-water pesticide exposure described above and counted subjects as exposed to an individual pesticide if the application rate was greater than 0 and unexposed if application rate was 0.

### Statistical analyses

We used multivariable unconditional logistic regression methods to calculate odds ratios (ORs) and 95% confidence intervals (CIs) to assess associations between possible exposure to pesticides from well water consumption and PD, adjusting for age (continuous), sex, education (< 12 years, 12 years, > 12 years), race/ethnicity (white, nonwhite), family history of PD (yes, no in first-degree relative), and smoking (never, former, current). In additional models, we adjusted for ambient exposure to the pesticide in order to examine the risk associated with consumption of potentially contaminated well water after controlling for the contribution of ambient exposure to the pesticide. Because adjusting for occupational exposure [using a job-exposure matrix (JEM) to estimate occupational pesticide exposure] did not appreciably change our results (ORs adjusted for occupational exposure to pesticides as estimated with the JEM were not more than 5% different than ORs not adjusted for occupational exposure), we opted for a more parsimonious model and did not adjust for occupational pesticide exposure in our final model. We analyzed separately each of the six individual pesticides listed above and also examined the total number of water-soluble, OP, and *N*-methylcarbamate pesticides and all pesticides together. We conducted tests and report *p*-values for trend using ordinal variables for pesticide use. All analyses used SAS version 9.1 (SAS Institute Inc., Cary, NC).

## Results

Study participants were predominantly Caucasian, > 65 years of age, and without a family history of PD (Table 2). Cases were slightly older than controls, were more likely to be male, and had completed fewer years of education. They were also more likely to have never smoked cigarettes.

In our population, 16.9% of all subjects reported private well water as their drinking water source some time during the 1974–1999 period. Cases were more likely to have consumed water from private wells than were controls during this period (Table 2) and reported drinking well water on average 4.3 (of the 26) years longer than controls (*p* = 0.02).

Consuming well water presumably contaminated at any level by one of the six pesticides that we examined separately was associated with PD, but only for diazinon did the 95% CI exclude the null value of 1 (Table 3). However, high levels of possible contamination resulted in 31–90% increases in risk compared with no well water contamination, with stronger associations seen for methomyl (OR = 1.67; 95% CI, 1.00–2.78), chlorpyrifos (OR = 1.87; 95% CI, 1.05–3.31), and propargite (OR = 1.92; 95% CI, 1.15–3.20). Only for diazinon in well water was the dose response reversed; that is, lower rather than higher levels of possible contamination with diazinon resulted in greater increases in risk of PD (Table 3). Adjusting for ambient pesticide exposures only slightly attenuated all well water pesticide effect estimates, with the largest change seen for propargite.

For all six pesticides examined individually, PD risk associated with possible contamination of well water and ambient exposures (19–75% relative increase) was greater than the risk associated with ambient exposures alone (15–57% relative risk increase; Table 4), also indicating that for most of these agents ambient exposure only still increased the risk.

Our combined estimates suggested that a higher number of water-soluble pesticides presumably contaminating well water increased the risk of PD (Table 5). Specifically, subjects exposed to ≥ 12 of the 26 pesticides included in the study or ≥ 10 water-soluble pesticides experienced a 66–68% greater relative risk. Also, subjects potentially exposed to the greatest number of OP pesticides in presumably contaminated well water experienced relative risk increases of similar magnitude (71%), and we found a trend with increasing numbers of OPs (*p*-trend = 0.04). However, possible contamination with multiple chemicals in the *N*-methyl carbamate class only slightly increased the risk of PD, if at all (the 95% CIs included the null), and we observed no trend with increasing numbers.

Because we noted greater estimated effect sizes for diazinon and chlorpyrifos when these pesticides were examined individually, we were concerned that the observed increases in PD risk for water-soluable OP classes of pesticides could have been mainly due to these two pesticides. Thus, we examined the water-soluble class after removing diazinon, and the OP class after removing both chlorpyrifos and diazinon, and found that the associations persisted. For the OPs, the OR for possible exposure in well water to 1 to 3 pesticides in this class was 1.07 (95% CI, 0.66–1.76), and for ≥ 4, the OR was 1.90 (1.07–3.03; *p*-trend = 0.04). For the water-soluble pesticides, the OR for possible well-water exposure to 1 to 8 pesticides in this class was 1.06 (95% CI, 0.65–1.72), and for > 8, the OR was 1.63 (95% CI, 0.98–2.70; *p*-trend = 0.08). These results suggest that the association between PD and the water-soluble or OP classes of pesticides investigated in this study is not dominated by 1 or 2 specific chemicals. Rather, exposure to a number of these types of pesticides in water may increase PD risk.

## Discussion

Our study population resides in a largely agricultural region of Central California with documented historical pesticide use since 1974. We found that potential exposure to pesticides from consumption of drinking water from private wells suspected to be contaminated with diazinon, methomyl, chlorpyrifos, propargite, or dimethoate in the 1974–1999 period was associated with an elevated risk of PD. High levels of possible well water contamination with methomyl, chlorpyrifos, and propargite resulted in approximately 70–90% increases in relative risk of PD compared with residents without such exposures from well water. For paraquat, the well water and ambient relative risk estimates were generally small and uninformative, which might be explained by our previous observation that exposure to paraquat may require coinciding maneb exposure to increase PD risk (Costello et al. 2009). Paraquat was examined in this study because of its interest to PD, and we recognized that its physical properties, including low water solubility and high adsorption, make it less likely to contaminate groundwater. Thus, we expected lower well water relative risk estimates for this pesticide compared with others examined in this study.

We also found that adjustment for ambient sources of pesticide exposure (i.e., inhalational, ingestional, or skin absorption routes of exposure) slightly attenuated but did not eliminate the observed associations for possible well water contamination. The PD relative risk associated with a combined exposure to pesticides in the environment and in presumably contaminated well water was greater than that associated with ambient exposure alone. These results suggest that, whereas exposure to the selected pesticides in the environment alone increases the relative risk of PD (20–50%), exposures from consumption of potentially contaminated well water may confer some additional, independent risk above ambient exposure.

Furthermore, consumption of well water presumably contaminated by a greater number of pesticides, specifically water-soluble pesticides or chemicals belonging to the OP class, further increased the risk of PD. Besides inhibiting acetylcholinesterase (Milatovic et al. 2006; Singh and Agarwal 1983), carbamate (Zhou et al. 2004) and OP (Sharma et al. 2005) pesticides are suspected to be involved in the etiologic pathway leading to PD, for example, by disturbing redox processes that inhibit antioxidant enzymes, thus enhancing lipid peroxidation and oxidative stress (Lukaszewicz-Hussain 2008) or inhibiting the proteasome or mitochondrial function in neurons.

Studies spanning two decades have examined the association between well water exposure and PD. Many of these studies were small, that is, included fewer than 100 cases (Marder et al. 1998; Morano et al. 1994; Smargiassi et al. 1998; Wang et al. 1993; Wechsler et al. 1991; Wong et al. 1991); all relied on self-reported well water consumption to define ever/never exposure groups, and none attempted to assess levels of general or specific pesticide contamination in well water. Most of these studies reported small relative increases in PD risk from ever being exposed to well water (ORs ranging from 1.02–2.8), and several found no associations (Chan et al. 1998; Golbe et al. 1990; Gorell et al. 1998; Hertzman et al. 1994; Marder et al. 1998; Tanner et al. 1999) or even reported protective associations (McCann et al. 1998; Wang et al. 1993), perhaps due to the absence of a toxic agent in the well water consumed in these study populations. Several studies included both rural and urban populations, and some of the wells may not have been located in areas where agricultural chemicals could have contaminated them. In fact, several authors noted that the introduction of chemicals to agricultural practices was a recent phenomenon in their study areas (De Michele et al. 1996; Liou et al. 1997; Tanner et al. 1989) and doubted that the period when pesticides may have contaminated well water would have been relevant to initiation of disease among the subjects they studied.

It is possible that one or more of the associations we report here do not reflect an etiologic contribution of the particular pesticide to PD risk, per se, but rather that the pesticide we suspected to have contaminated the well water consumed by our population acted as a surrogate measure for another unidentified pesticide, that is, other pesticides in use that are strongly correlated with the pesticide we examined. Exposure to mixtures of chemicals is a problem inherent in the assessment of exposure in humans. Among the 26 pesticides purposefully selected for our study, several were generally coapplied. For the six pesticides we individually examined, for example, among subjects who were ambiently exposed to chlorpyrifos at their residences, 80% were also exposed to diazinon and 91% to paraquat; of subjects ambiently exposed to paraquat, 73% were also exposed to diazinon, 82% to methomyl, and 80% to propargite. Thus, it was also impossible to estimate the effects for all of the six pesticides together in the same model, that is, to estimate the effect for one chemical while adjusting for all others. To avoid issues of multiple testing as much as possible while still evaluating the most relevant water contaminants, we restricted the pesticides selected for analyses to those with a high probability of being found in groundwater, based on their physical properties/chemistry and using the CADPR list of pesticides previously detected in groundwater or recognized as potential groundwater contaminants as a guide. Nevertheless, we cannot rule out that some of our findings might be due to chance.

It is also possible that well water in rural locations may be contaminated with multiple agricultural and industrial agents and metals, in addition to pesticides. DeMichele et al. (1996) noted that farming practices and exposure to chemicals vary from area to area as a possible explanation for diverging results in the literature. To our knowledge, no previous study of PD has estimated pesticide residue contamination historically in drinking water; we are the first to implement a semiquantitative approach to estimating pesticide exposure. Our well water pesticide exposure estimates do not exclusively reflect exposure from water ingestion alone because the suspected contamination was derived from data on applications in proximity of wells supplying water to residences, and these same chemicals were likely also air and soil contaminants. However, we did adjust and possibly may have even overadjusted for ambient pesticide exposure in our models, and found minimal attenuations in our well water risk estimates; that is, the associations for most chemicals remained after adjustment. An additional limitation is that our models for water contamination did not take into account some geologic factors such as soil quality, groundwater depth, and direction of groundwater flow that could influence the likelihood that a pesticide reaches the water drawn from private wells. Thus, our pesticide well water exposure estimates may not completely reflect actual levels of exposure to pesticides from consuming well water.

Our study is unique among those that have examined PD risk from well water consumption in that we used existing historical California PUR data, which we combined with land-use maps to derive pesticide application rates for the study area over an extended period. Thus, our well water pesticide exposure measure is an estimate derived from our GIS models; we did not sample well water to directly measure actual current or historical pesticide levels. Rajput et al. (1987) found no differences in concentrations of several metals in samples taken from wells that provided drinking water to PD cases and controls in Canada. However, given that well water sampling in that study was performed at the time of PD diagnosis, measured levels may not have accurately reflected contamination during the critical exposure window for PD years or decades before diagnosis.

In an attempt to validate our model for well water pesticide contamination, we obtained domestic well water sample data from the CADPR; the agency analyzed these samples for multiple chemicals on a nonroutine basis for nearly three decades (1980 to present). The current database contains > 95,000 records from approximately 9,300 domestic wells located in about 4,100 township range sections throughout California, and lists as detected about 200 possible pesticide active ingredients and breakdown products. Sampling, however, did not follow any standardized schedule and was not performed randomly for all private wells. When we cross-referenced the CADPR data with addresses for our study subjects, data on testing for and detecting pesticides were available for wells located in the same township range section as a residence for no more than 20 cases and 17 controls and for a total of nine pesticides. For two of the more common pesticides identified (simazine and diuron), our GIS models had identified approximately 7.5% of the study population as being ambiently exposed. We cross-tabulated CADPR detections (yes, no/nondetect) with our ambient exposure measures (yes, no) and found moderate concordances. For simazine, our model-predicted exposures corresponded with the CADPR detections 63.5% of the time; for diuron, it was 65%. However, it is not possible to know whether the wells sampled by CADPR were the same wells from which study subjects had drawn their drinking water.

Our study represents a significant improvement over other previous studies (Ascherio et al. 2003; Marder et al. 1998; Morano et al. 1994; Smargiassi et al. 1998; Wang et al. 1993; Wechsler et al. 1991; Wong et al. 1991) in that we did not have to rely on study subjects’ recall of their own pesticide use to derive exposure estimates, a procedure criticized for its potential to introduce differential exposure misclassification bias if cases and controls recall differently. An additional strength of our present study is that all of our PD diagnoses were clinically confirmed by a study movement disorder specialist, and thus we expect our results to be only minimally affected by disease misclassification.

In conclusion, our study, the first of its kind to apply a semiquantitative approach to estimating pesticide exposure in well water, contributes evidence that consumption of well water potentially contaminated with pesticides may play a role in the etiology of PD.

## Correction

In Table 2 of the manuscript originally published online, values for “Ever had a private well” were incorrect. They have been corrected here.

## Figures and Tables

**Table 1:** Pesticides selected for study, cumulative application rates during the period 1974–1999^a^ in a 500-m buffer around residences with a private well reported as water supply, reason for selection, and water solubility.

Pesticide	Chemical family/use	Median (range) pesticide application rate (lb/acre)^b^	Reason^c^	Water soluble
Triflumizole	Azole/fungicide	6.51 (0.56–31.9)	3	Yes
Vinclozolin	Dicarboximide/fungicide	1.92 (< 0.01–8.1)	3	Yes
Maneb	Dithiocarbamate/fungicide	11.24 (1.52–169.1)	1	No
Aldicarb	*N*-Methyl carbamate/insecticide	11.18 (0.19–221.0)	3	Yes
Methomyl	*N*-Methyl carbamate/insecticide	8.26 (< 0.01–302.8)	3	Yes
Carbofuran	*N*-Methyl carbamate/insecticide, nematicide	3.88 (< 0.01–52.2)	3	Yes
Carbaryl	*N*-Methyl carbamate/insecticide, nematicide, plant growth regulator	44.25 (0.01–756.0)	3	Yes
Dicofol	Organochlorine/acaricide	14.38 (< 0.01–239.2)	4	No
Acephate	OP/insecticide	4.97 (0.06–34.9)	3	Yes
Azinphos-methyl	OP/insecticide	34.76 (0.36–671.7)	3	Yes
Chlorpyrifos	OP/insecticide	28.97 (< 0.01–884.3)	4	No
Diazinon	OP/insecticide	44.31 (0.07–2493.0)	3	Yes
Dimethoate	OP/insecticide	14.42 (< 0.01–437.1)	3	Yes
Oxydemeton-methyl	OP/insecticide	1.37 (0.06–70.9)	3	Yes
Parathion	OP/insecticide	57.45 (0.01–1412.3)	3	Yes
Trichlorfon	OP/insecticide	7.14 (0.07–42.7)	4	Yes
Disulfoton	OP/insecticide, nematicide	8.33 (< 0.01–174.5)	3	Yes
Phorate	OP/insecticide, nematicide	11.43 (0.12–207.8)	3	Yes
Permethrin	Pyrethroid/insecticide	0.69 (< 0.01–292.3)	1	No
Paraquat	Quaternary ammonium/herbicide	19.38 (0.01–1683.5)	1	Yes
Chlorothalonil	Substituted benzene/fungicide	18.86 (0.02–1265.4)	3	No
Diuron	Substituted urea/herbicide	53.59 (0.69–788.4)	2	Yes
Propargite	Sulfite ester/acaricide	34.84 (< 0.01–2116.8)	4	No
Simazine	Triazine/herbicide	29.83 (1.15–632.8)	2	Yes
Linuron	Urea/herbicide	3.93 (0.09–55.9)	3	Yes
Chloropicrin	Unclassified/fumigant, nematicide	6.83 (< 0.01–370.9)	3	No

a Using data on cumulative pesticide application rates for years during this period when pesticides were applied.

b Based on the distribution among controls.

c 1, of interest to PD; 2, detected by CADPR in groundwater; 3, identified by CADPR or other agencies as potential groundwater contaminant; 4, high relative runoff or leaching potential.

**Table 2:** Characteristics of Parkinson’s Environment and Gene Study population.

Characteristic	Cases (*n* = 368)	Controls (*n* = 341)	OR (95% CI)
Age [years (mean ± SD)]	69.6 ± 10.3	67.6 ± 11.4	1.02 (1.00–1.03)
Female sex	161 (43.8)	165 (48.4)	0.83 (0.62–1.12)
Race			
White	314 (85.3)	292 (85.6)	Reference
Nonwhite	54 (14.7)	49 (14.4)	1.03 (0.68–1.56)
Black	2 (0.5)	11 (3.2)	
Latino	46 (12.5)	29 (8.5)	
Asian	4 (1.1)	8 (2.4)	
Native American	2 (0.5)	1 (0.3)	
Education (years)			
< 12	68 (18.5)	38 (11.1)	2.14 (1.38–3.32)
12	100 (27.2)	64 (18.8)	1.87 (1.30–2.69)
> 12	200 (54.4)	239 (70.1)	Reference
Smoking status			
Current	22 (5.9)	34 (10.0)	0.48 (0.27–0.86)
Former	151 (41.0)	161 (47.2)	0.70 (0.52–0.96)
Never	195 (53.0)	146 (42.8)	Reference
First-degree relative with PD	55 (15.0)	38 (11.1)	1.40 (0.90–2.18)
Ever had private well water^a^	259 (70.4)	234 (68.6)	1.21 (0.82–1.80)
Total years used well water (mean ± SD)^b^	18.3 ± 9.3	14.0 ± 10.0	

Values are number (%) except where indicated.

a Lifetime.

b Years represent 1974–1999 data.

**Table 3:** Relative risk of PD from potential exposure to individual pesticides in well water.

	Exposure vs. no exposure			Low or high exposure vs. no exposure^a^			
Pesticide	Exposure level	Cases/controls	OR^b^ (95% CI)	Exposure level	Cases/controls	OR^b^ (95% CI)	OR^c^ (95% CI)
Diazinon	None	295/300	1.0 (reference)	None	295/300	1.0 (reference)	1.0 (reference)
	Any	73/41	1.58 (1.03–2.43)	Low	45/21	2.00 (1.14–3.50)	2.00 (1.14–3.50)
				High	28/20	1.16 (0.62–2.14)	1.14 (0.53–2.43)

Dimethoate	None	290/290	1.0 (reference)	None	290/290	1.0 (reference)	1.0 (reference)
	Any	78/51	1.41 (0.94–2.11)	Low	33/26	1.28 (0.74–2.24)	1.29 (0.74–2.24)
				High	45/25	1.53 (0.90–2.62)	1.47 (0.83–2.58)

Methomyl	None	290/288	1.0 (reference)	None	290/288	1.0 (reference)	1.0 (reference)
	Any	78/53	1.28 (0.85–1.91)	Low	26/26	0.86 (0.48–1.56)	0.87 (0.48–1.58)
				High	52/27	1.67 (1.00–2.78)	1.40 (0.80–2.43)

Chlorpyrifos	None	301/300	1.0 (reference)	None	301/300	1.0 (reference)	1.0 (reference)
	Any	67/41	1.45 (0.94–2.24)	Low	25/21	1.05 (0.56–1.96)	1.05 (0.56–1.96)
				High	42/20	1.87 (1.05–3.31)	1.81 (1.00–3.30)

Propargite	None	291/288	1.0 (reference)	None	291/288	1.0 (reference)	1.0 (reference)
	Any	77/53	1.31 (0.88–1.96)	Low	22/27	0.73 (0.40–1.34)	0.73 (0.40–1.34)
				High	55/26	1.92 (1.15–3.20)	1.94 (1.07–3.52)

Paraquat	None	289/281	1.0 (reference)	None	289/281	1.0 (reference)	1.0 (reference)
	Any	79/60	1.10 (0.75–1.63)	Low	32/30	0.89 (0.52–1.54)	0.89 (0.51–1.54)
				High	47/30	1.31 (0.79–2.17)	1.26 (0.72–2.20)

a Based on median value in controls to distinguish between low and high exposure levels.

b Adjusted for age, race, sex, education, and family history of PD.

c Adjusted for above covariates and ambient pesticide exposure.

**Table 4:** Relative risk of PD from potential inhalation and ingestion of pesticides.

Pesticide/exposure	Cases/controls	OR^a^ (95% CI)
Diazinon		
Unexposed	165/188	1.0 (reference)
Ambient pesticide only	130/112	1.29 (0.92–1.81)
Ambient and well water	73/41	1.75 (1.12–2.76)
Dimethoate		
Unexposed	150/180	1.0 (reference)
Ambient pesticide only	140/110	1.57 (1.12–2.22)
Ambient and well water	78/51	1.72 (1.12–2.65)
Methomyl		
Unexposed	147/165	1.0 (reference)
Ambient pesticide only	143/123	1.23 (0.87–1.72)
Ambient and well water	78/53	1.41 (0.91–2.18)
Chlorpyrifos		
Unexposed	186/210	1.0 (reference)
Ambient pesticide only	115/90	1.42 (1.00–2.01)
Ambient and well water	67/41	1.63 (1.04–2.57)
Propargite		
Unexposed	152/164	1.0 (reference)
Ambient pesticide only	139/124	1.24 (0.88–1.75)
Ambient and well water	77/53	1.45 (0.94–2.23)
Paraquat		
Unexposed	131/140	1.0 (reference)
Ambient pesticide only	158/141	1.15 (0.82–1.62)
Ambient and well water	79/60	1.19 (0.77–1.83)

a Adjusted for age, race, sex, education, and family history of PD.

**Table 5:** Relative risk of PD from potential exposure^a^ to pesticides in well water by chemical family or water solubility of pesticide.

Pesticide family or water solubility/number exposed	Cases/controls	OR^b^ (95% CI)	*p*-Trend
Water soluble pesticides (*n* = 19)			
0	273/275	1.0 (reference)	
1–9	43/39	1.03 (0.63–1.67)	
≥ 10	52/27	1.68 (1.01–2.81)	0.07

OPs pesticides (*n* = 10)			
0	272/277	1.0 (reference)	
1–4	36/33	1.03 (0.61–1.74)	
≥ 5	60/31	1.71 (1.06–2.76)	0.04

*N*-Methyl carbamate (*n* = 4)			
0	281/282	1.0 (reference)	
1–2	44/30	1.35 (0.81–2.26)	
≥ 3	43/29	1.24 (0.73–2.08)	0.26

All pesticides (*n* = 26)			
0	270/273	1.0 (reference)	
1–11	36/35	0.95 (0.57–1.60)	
≥ 12	62/33	1.66 (1.04–2.66)	0.06

a Based on exposed/unexposed classification.

b Adjusted for age, race, sex, education, and family history of PD.
